# Supplementary material for: Genetic control of meristem arrest and life span in Arabidopsis by a FRUITFULL-APETALA2 pathway
Source: Nat Commun. 2018 Feb 8;9:565. doi: 10.1038/s41467-018-03067-5 (PMC5805735; doi:10.1038/s41467-018-03067-5)
Supplement: Supplementary file 1 — Supplementary Information [file 41467_2018_3067_MOESM1_ESM.pdf]

Supplementary Information.

Genetic control of meristem arrest and life span in *Arabidopsis* by a *FRUITFULL-APETALA2* pathway

Balanzà et al.

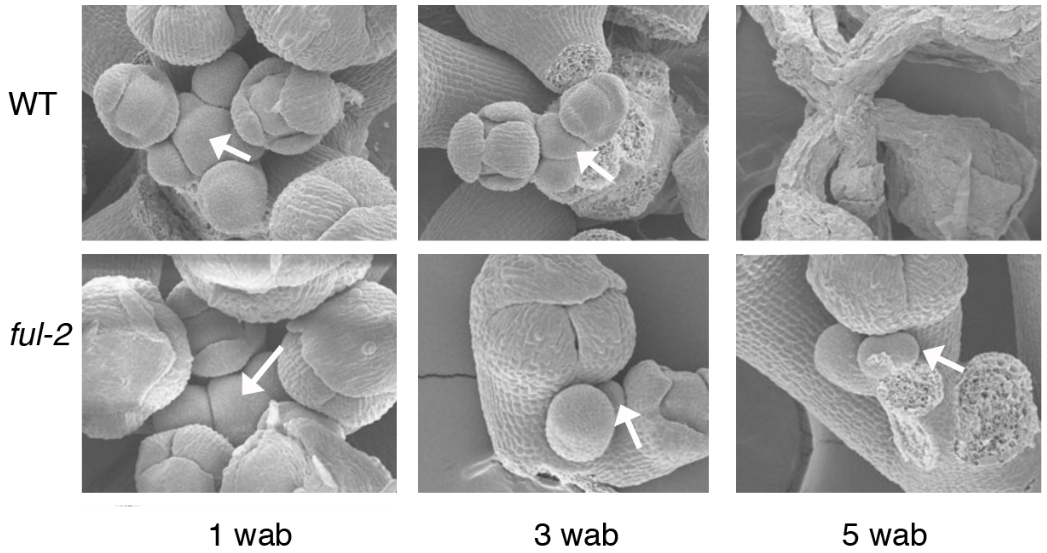

Supplementary Figure 1.

Shoot apical meristem size decreases with plant age. SEM micrograph of the SAM of wildtype Col-0 and *ful-2* mutants at different stages of development show a consistent and similar decrease in SAM size in both backgrounds. However, while in wildtype plants at 5 weeks after bolting (wab), the SAM is no longer visible, in *ful* mutants the SAM still is discernible although with very small size. White arrows mark SAM in all panels.

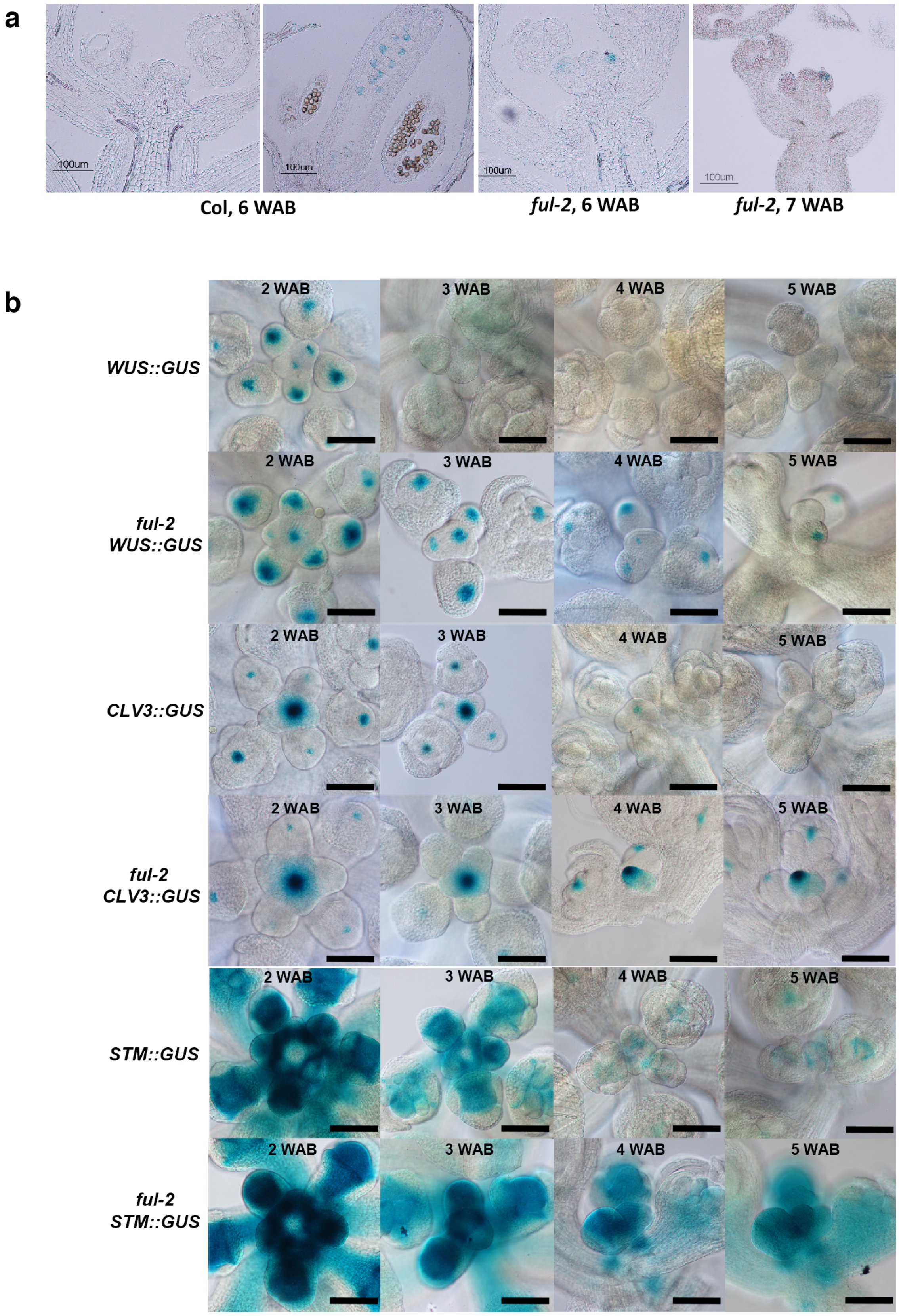
a
100um
100um
100um
100um
Col, 6 WAB
ful-2, 6 WAB
ful-2, 7 WAB
b
2 WAB
3 WAB
4 WAB
5 WAB
WUS::GUS
ful-2
WUS::GUS
CLV3::GUS
ful-2
CLV3::GUS
STM::GUS
ful-2
STM::GUS

Supplementary Figure 2.

(a) WUS::GUS expression at 6 or 7 weeks after bolting. Sections of Col or *ful-2* inflorescences stained to detect WUS::GUS activity. WUS::GUS is no longer detected in SAM of wildtype Col apices, while still present in ovules of developing flowers in these same Col inflorescences and in *ful-2* SAMs. (b) **Time course of WUS::GUS, CLV3::GUS and STM::GUS in wildtype or *ful-2* inflorescences**. WUS::GUS expression is no longer detected in wildtype SAMs around 4 weeks after bolting (WAB), while still present in *ful-2* inflorescences. CLV3::GUS activity decreases with time and disappears in wildtype SAMs around 5 wab, while is still present in *ful-2*. STM::GUS expression is maintained for longer both in wildtype and *ful-2* backgrounds, although at higher levels in *ful-2* mutants.

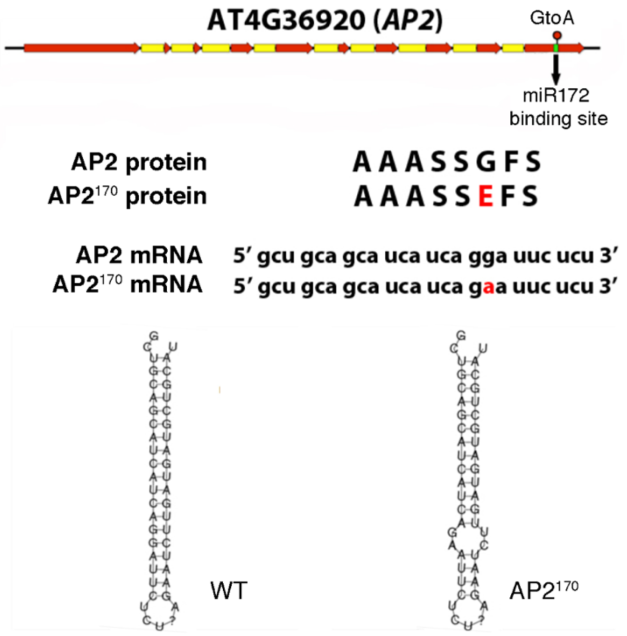

Free energy for the duplex dG=Kcal/mol			
	**miR172a**	**miR172b**	**miR172c**
**AP2 wt GGA**	-39.68	-42.13	-39.78
**AP2^{170} GAA**	-33.22	-35.67	-33.32

Supplementary Figure 3.

A polymorphism in the *AP2* CDS causes the phenotypic alterations in L170.2 line. A G to A transition in the CDS of the *AP2* gene causes a Glycine to Glutamate change in the AP2 protein as well as a mismatch in the miR172 recognition site. This change is predicted to affect the stability of the miR172/AP2 mRNA alignment (according to the prediction software available at Weigelworld bioinformatics portal).

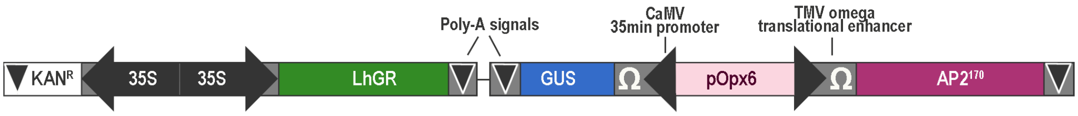

Supplementary Figure 4.

Schematic representation of the construct used to generate the inducible version of AP2^{170}

A dexamethasone-inducible transactivating system was used to induce the expression of AP2^{170} upon dexamethasone treatment in plants that had already undergone GPA. This system consists of two components. The first unit contains an artificial transcription factor (LhGR) comprising the DNA-binding domain of the lac repressor, the activation domain of GAL4 and the ligand-binding domain of the rat glucocorticoid receptor (GR LBD). The second unit consists of a six-multimerised binding site for lac, fused to the minimal 35S promoter at both ends, forming a bidirectional promoter that drives the expression of the β- glucuronidase (GUS) gene and the gene of interest.

a

locus	name	peak enrichment score
AT2G17950	WUS	NS (< 7.99)
AT2G28056	MIR172A	NS (< 7.99)
AT2G28550	TOE1	42.70
AT2G39250	SNZ	18.14
AT3G11435	MIR172C	NS (< 7.99)
AT3G55512	MIR172D	NS (< 7.99)
AT4G36920	AP2	95.23
AT5G04275	MIR172B	NS (< 7.99)
AT5G59505	MIR172E	NS (< 7.99)
AT5G67180	TOE3	38.67

b

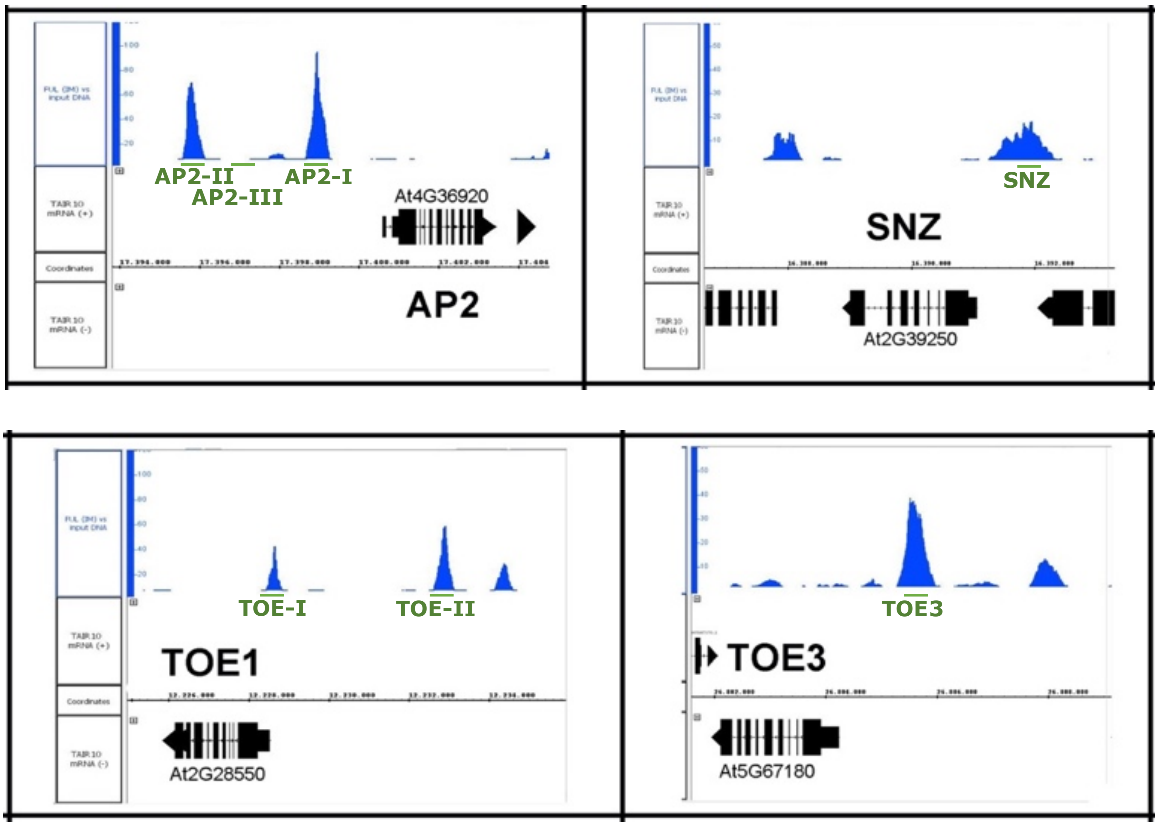

Supplementary Figure 5.

FUL binds to the *AP2*, *TOE1*, *TOE3* and *SNZ* promoters. (A) Shortlist of binding sites identified in a ChIP-seq experiment with *ap1 cal* pFUL::FUL:GFP inflorescence meristems (GEO acc. number GSE108455). The peak enrichment scores are based on the maximum enrichment identified in a region from 3kb upstream until 1kb downstream of the gene. Significant peaks, with a FDR (false discovery rate) < 0.05, have an enrichment score higher than 7.99. (B) Location of the FUL binding peaks in the promoters of *AP2*, *SNZ*, *TOE1* and *TOE3* displayed with the Integrated Genome Browser. The orange lines indicate the fragment positions used for ChIP-PCR, Luc assays (AP2) and EMSA.

a

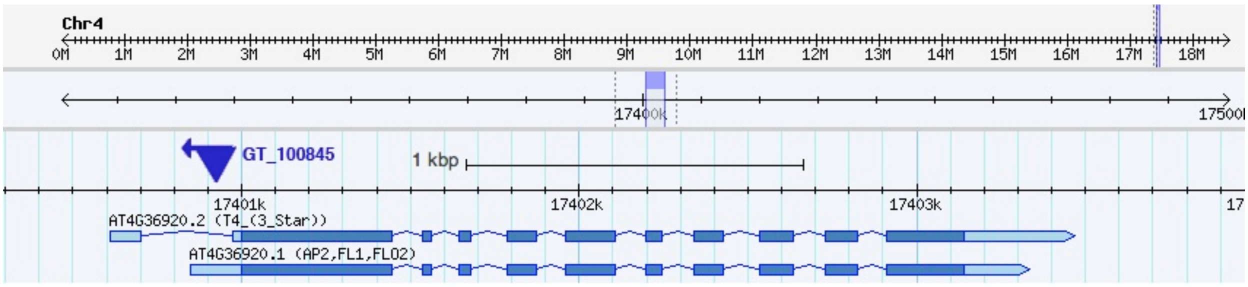

b

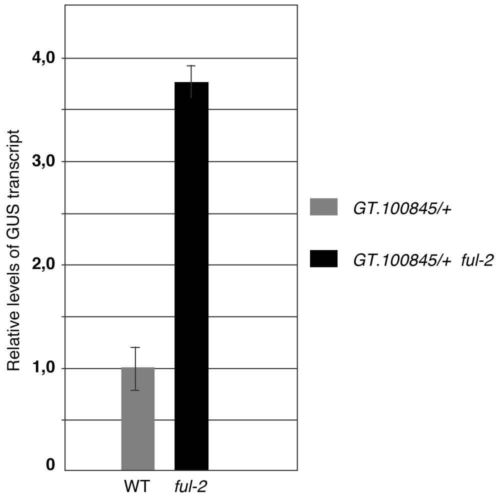

Supplementary Figure 6.

GUS mRNA levels in the Gene Trap line GT.100845 are elevated in *ful* mutants. (A) Schematic representation of the insertion site of the transposon in the GT_100845 line according to the information provided in the TAIR webpage (www.arabidopsis.org). (B) qRT-PCR analyses of GUS expression were made in whole inflorescences from plants heterozygous for the transgene in either wildtype Col-0 background or in *ful-2* mutant background

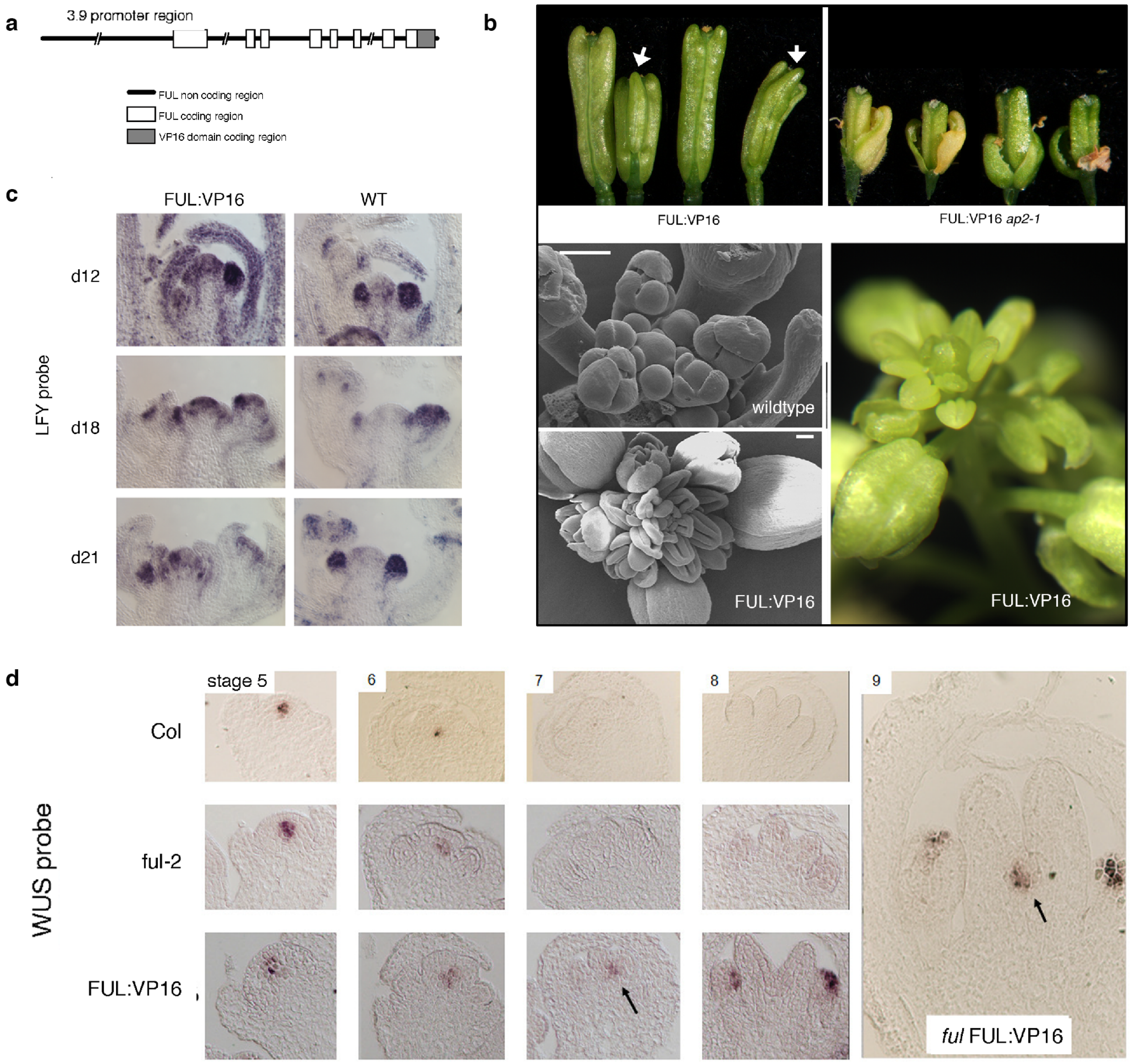

Supplementary Figure 7.

Structure of the pFUL::FUL:VP16 transgene and the fruit phenotypes of transgenic plants. (a) Schematic representation of the FUL:VP16 allele. (b) The pFUL::FUL:VP16 transgene causes mild indeterminacy defects when introduced in a wildtype background, with frequent production of extra valves in fruits (arrows in top panel). In addition, fruits are shorter, wider and heart-shaped. The floral indeterminacy defects caused by FUL:VP16 are mostly suppressed in the *ap2-1* background, where extra valves are not observed. In addition, FUL:VP16 plants cause the SAM to differentiate in a highly indeterminate flower, similar to that of 35S::AP2m3 lines. (c) The apical indeterminate flower of FUL:VP16 lines ectopically expresses *LFY* as detected by in situ hybridization of *LFY* RNA. In the figure, a time course through inflorescence development compares *LFY* expression in WT and FUL:VP16 plants. Days after germination are indicated. (d) *WUS* expression is detected at more advanced floral stages in FUL:VP16 and FUL:VP16 *ful* flowers (arrows).

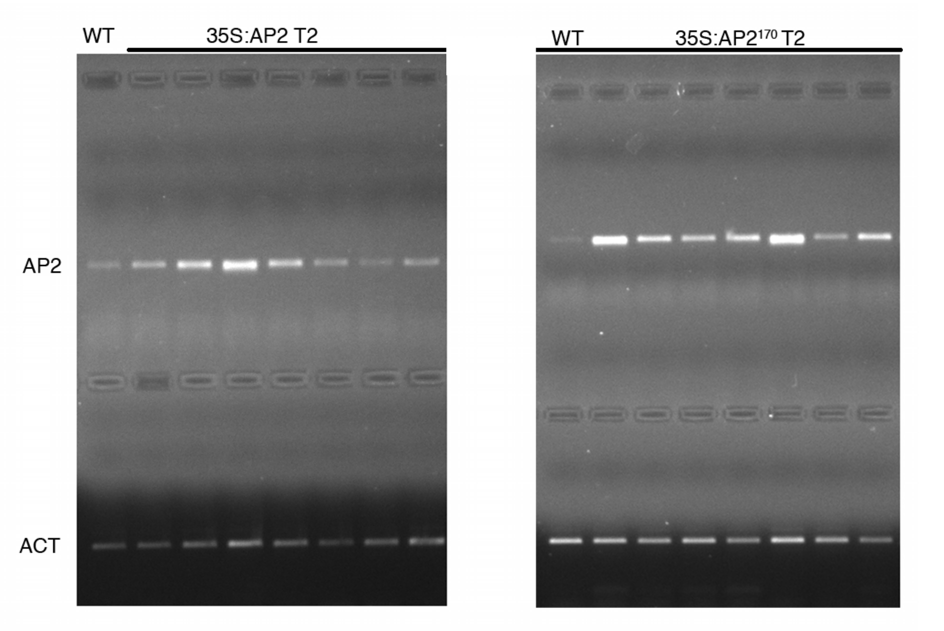

Supplementary Figure 8.

Expression of transgenes in 35S::AP2 and 35S::AP2^{170} lines. To confirm that the constructs used to overexpress AP2 or AP2^{170} were functional, we performed a semiquantitative PCR on cDNA obtained from pooled seedlings (where miR172 levels are low) of randomly selected T2 families from 35S::AP2 or 35S::AP2^{170}. Total cDNA was quantified and equal amounts were used per PCR reaction. Bands on the top part of the gel correspond to the AP2 gene and bands in the lower part of the gel to the reference gene ACTIN, both after 22 cycles of amplification. As it could be expected, given the partial resistance of AP2^{170} to miR172 degradation, the 35S:AP2^{170} lines show higher relative expression of AP2.
