# Supplementary material for: Genetic control of meristem arrest and life span in Arabidopsis by a FRUITFULL-APETALA2 pathway
Source: Nat Commun. 2018 Feb 8;9:565. doi: 10.1038/s41467-018-03067-5 (PMC5805735; doi:10.1038/s41467-018-03067-5)
Supplement: Supplementary file 3 — Description of Additional Supplementary Files [file 41467_2018_3067_MOESM3_ESM.pdf]

Description of Supplementary Files

File Name: Supplementary Data 1
Description: Primers used in this study for all experiments including cloning, ChIP-PCR, EMSA and expression studies by qRT-PCR and RT-PCR.
